# A Quantitative EEG Toolbox for the MNI Neuroinformatics Ecosystem: Normative SPM of EEG Source Spectra

**DOI:** 10.3389/fninf.2020.00033

**Published:** 2020-08-07

**Authors:** Jorge Bosch-Bayard, Eduardo Aubert-Vazquez, Shawn T. Brown, Christine Rogers, Gregory Kiar, Tristan Glatard, Lalet Scaria, Lidice Galan-Garcia, Maria L. Bringas-Vega, Trinidad Virues-Alba, Armin Taheri, Samir Das, Cecile Madjar, Zia Mohaddes, Leigh MacIntyre, CHBMP, Alan C. Evans, Pedro A. Valdes-Sosa

**Affiliations:** ^1^The Clinical Hospital of Chengdu Brain Sciences Institute, University of Electronic Science and Technology of China UESTC, Chengdu, China; ^2^McGill Centre for Integrative Neuroscience (MCIN), Ludmer Centre for Neuroinformatics and Mental Health, Montreal Neurological Institute (MNI), McGill University, Montreal, QC, Canada; ^3^Cuban Neuroscience Centre, Havana, Cuba

**Keywords:** Statistical Parametric Mapping, qEEGt, CBRAIN, EEG tomography, quantitative EEG, open science

## Abstract

The Tomographic Quantitative Electroencephalography (qEEGt) toolbox is integrated with the Montreal Neurological Institute (MNI) Neuroinformatics Ecosystem as a docker into the Canadian Brain Imaging Research Platform (CBRAIN). qEEGt produces age-corrected normative Statistical Parametric Maps of EEG log source spectra testing compliance to a normative database. This toolbox was developed at the Cuban Neuroscience Center as part of the first wave of the Cuban Human Brain Mapping Project (CHBMP) and has been validated and used in different health systems for several decades. Incorporation into the MNI ecosystem now provides CBRAIN registered users access to its full functionality and is accompanied by a public release of the source code on GitHub and Zenodo repositories. Among other features are the calculation of EEG scalp spectra, and the estimation of their source spectra using the Variable Resolution Electrical Tomography (VARETA) source imaging. Crucially, this is completed by the evaluation of z spectra by means of the built-in age regression equations obtained from the CHBMP database (ages 5–87) to provide normative Statistical Parametric Mapping of EEG log source spectra. Different scalp and source visualization tools are also provided for evaluation of individual subjects prior to further post-processing. Openly releasing this software in the CBRAIN platform will facilitate the use of standardized qEEGt methods in different research and clinical settings. An updated precis of the methods is provided in Appendix I as a reference for the toolbox. qEEGt/CBRAIN is the first installment of instruments developed by the neuroinformatic platform of the Cuba-Canada-China (CCC) project.

## Introduction

Electroencephalography (EEG) is one of the oldest, most useful, and widely deployable methods to study normal and pathological brain function. It is characterized by its sensitivity and exquisite temporal resolution (Niedermeyer et al., 2010). Unfortunately, this type of physiological measurement fell out of favor in research and clinical applications a few decades ago, “eclipsed” by the new neuroimaging techniques (Single Photon Emission Tomography—SPECT, Positron Emission Tomography—PET, and Functional Magnetic Resonance Imaging—fMRI) that were deemed to have true 3D spatial resolution. In fact, it was affirmed that EEG was not even an imaging modality, or in any case, was one with a very poor spatial resolution. This neglect of EEG has been detrimental to translational Neurotechnology.

The negative perception of electrophysiology is now being reversed. Fundamental to this is the development of EEG Source Imaging (ESI) that has achieved considerable maturity (Michel et al., 2004) by leveraging Bayesian estimation prior information about source localizations and connectivity (Wang et al., 2019). As recently reviewed in Babiloni et al. (2019b) ESI is currently an active area of clinical research.

A convergent, but separate, strand of EEG clinical research has been known as “quantitative analysis of EEG” (qEEG) (John et al., 1977; Pardoux, 2008). For a detailed history see Hernandez-Gonzalez et al. (2011). In its most widely used form, the Tomographic Quantitative Electroencephalography (qEEGt) technique extracts frequency specific information about normal and abnormal brain states via the EEG frequency spectrum at scalp electrodes. It then tests for compliance of each electrode and frequency bin (or band) to a normative, most commonly by transformation of each log spectral value to a z transform with respect to an age specific mean and standard deviation. This has been shown to be a useful preprocessing step for either visual inspection or to use multivariate methods to detect and classify brain pathology (Fernández-Bouzas et al., 1995; Hernandez-Gonzalez et al., 2011; Nunez et al., 2019). z values are displayed as topographic maps on the scalp as statistical tests for deviations from normative data. This “significance probability mapping” (spm) inspired developments in Neuroimaging. In fact, SPM (acronym in capitals, Statistical Parametric Mapping) (Friston et al., 1995) owes its acronym to this type of spatial display of statistical tests, but for 3D neuroimages from other imaging modalities (PET, MRI, fMRI). The transition from EEG spm to SPM required to provide a 3D extension of qEEG by means of ESI.

This transformation of qEEG spm to SPM was originally achieved in 2001 (Bosch-Bayard et al., 2001). In this work:

a) The Variable Resolution Electrical Tomography (VARETA) electrophysiological source imaging method was used to obtain source spectra and their log transforms over a defined grid of voxels with high frequency resolution (Szava et al., 1994).
b) Statistical Parametric maps of z-sores for the log source spectra were obtained for each voxel and each frequency bin. Each z score is obtained by subtracting an age-dependent mean and dividing by the age-dependent standard deviation.
c) These age dependent means and standard deviations are embodied in a set of age regression equations for each voxel and each frequency bin.

This “normative SPM of EEG source spectra” is what we have termed “quantitative EEG tomography” or qEEGt. It is essential to note that the current qEEGt toolbox was based on the first wave (1988–2003) of the Cuban Human Brain Mapping Project (CHBMP) (Hernandez-Gonzalez et al., 2011), which acquired the EEG of 211 subjects aged 5–87, randomly selected from the general population. Due to the lack of an individual Magnetic Resonance Image (iMRI) for each subject, an “average head model” was used (Evans et al., 1993). The validity and accuracy of this approach to calculate an approximate lead field has been described elsewhere (Valdés-Hernández et al., 2009). Rather than being a drawback, this use of an approximate head model for ESI has proven to be a valuable instrument in settings which preclude the use of iMRIs (Bosch-Bayard et al., 2012). qEEGt has thus been acknowledged as the first application of Statistical Parametric Mapping to electrophysiology (Friston, 2007, p. 8: “The MEG-EEG years”). The full formal specification for qEEGt is provided in Appendix I.

In view of these developments it is surprising that most of the major brain initiatives such as the UK Biobank (www.ukbiobank.ac.uk), ADNI (adni.loni.usc.edu), ABCD (https://abcdstudy.org) have no electrophysiological component. Fortunately the Human Connectome Project (http://www.humanconnectomeproject.org/) and the CAMCAN (www.cam-can.org) project have at least included MEG data collections, thus providing temporal resolution equivalent to EEG. In Canada, the Brain-Code project (https://braininstitute.ca/research-data-sharing/brain-code) has been launched, which is an informatics platform that hosts several biological EEG/MEG data across a growing list of brain pathologies that is shared by over 20 institutions in Ontario, and around 120 researchers. This initiative supports the EEG working group of the Canadian Biomarker Integration Network in Depression (CAN-BIND) that elaborates guidelines for EEG recording and processing standardization (Farzan et al., 2017).

We believe that this situation is partly due to the lack of open-access, structured pipelines, embedded in a major neuroimaging Neuroinformatics platform. While there exist now many different source imaging methods (Vega-Hernández et al., 2008), current packages for this purpose do not provide SPM for the comparison of spectral parameters against validated, population based normative data. Those few packages which in effect provide this functionality, do not make their high dimensional set of regression equations publicly available but rather keep them proprietary.

In this paper we provide an open-access pipeline integrating the qEEGt analysis toolbox developed at the Cuban Neuroscience Center (CNEURO) with a major processing portal for deployment of advanced neuroimaging pipelines: the Canadian Brain Imaging Research Platform (CBRAIN) (Sherif et al., 2014) and the Longitudinal Online Research and Imaging System (LORIS) (Das et al., 2012). Not only can this pipeline be accessed via CBRAIN but the exact version of the qEEGT toolbox, which includes the VARETA source imaging method, the full set of regression equations with regard to age, as well as the procedures for calculation of z-spectra are also publicly available in Github: https://github.com/CCC-members/QEEGT-Toolbox (doi: https://doi.org/10.5281/zenodo.3745563). Making the code available also facilitates its use by users who may want to merge the qEEGT with their own tools or want to integrate it with other tools like EEGLAB via plugins. This last choice may be attractive for EEGLAB users since EEGLAB can read many different EEG formats. In the future it would be possible creating text files from different EEG formats loaded by EEGLAB to be used by the qEEGT toolbox widening the scope of the present contribution.

For those interested in comparing the formulation described in Appendix I for the frequency domain VARETA (FD-VARETA), with the code provided in the github and Zenodo repositories, the major part of its implementation can be found among lines 1090–1230 and then from lines 1719–1804 of the github/Zenodo code. In this version we only implemented part of the FD-VARETA methodology. Some quantities were pre-calculated and assumed constant since their calculation is time-consuming. It is explained in Appendix I. A modern full implementation of FD-VARETA, that fulfills the formulation described in Appendix I can be found in https://github.com/CCC-members/BC-VARETA_Toolbox. In that toolbox, the equivalent version to the formulation of the present paper is the ridge penalty. A more advanced methodology also included in that toolbox is based on the graphical lasso penalty.

## Normative Database

A feature of this toolbox is that it includes normative data which allows the calculation of univariate measurement of deviation from normality of the log EEG spectra both at the scalp and at the sources. To our knowledge, this is the first qeeg toolbox that makes freely available this type of information.

The normative data provided with this toolbox were obtained from the first wave Cuban Human Brain Mapping project (CHBMP) (Hernandez-Gonzalez et al., 2011). They comprise age regression coefficients for all scalp channels and sources in the frequency range of 0.39–19.11 Hz, with a sampling resolution of 0.39 Hz. The age range goes from 5 to 87 years old of a sample of 211 normal subjects obtained from the normal Havana population.

Age dependent regressions were calculated for the Eyes Closed, Eyes Open, and Hyperventilation states. The sample was selected from Havana population using a quasi-random algorithm, to guarantee a balanced age representation. Strict clinical criteria were followed to eliminate from the sample subjects who were not functionally healthy.

The subjects were recorded during the morning to guarantee the state of wakefulness. The following instructions were given prior to the EEG recording and checked for just before the session: (a) to go to bed before 11 pm the night before and sleep for at least 8 h; (b) to abstain from alcohol, coffee, black tea, chocolate or soda the day before; (c) to and to have abnormal breakfast in the morning. Additionally, before starting the recording at the clinic they were offered a snack to avoid prolonged fasting period.

## Cbrain Overview

CBRAIN is a Montreal Neurological Institute (MNI) initiative developed to address the storage and processing needs driven by the unprecedented growth of neuroimaging data and distributed computing infrastructure. It has been developed as a collaborative high-performance computing (HPC) portal enabling efficient processing of high volumes of data across national networks such as the Compute Canada clusters. The platform allows researchers to perform computationally intensive analyses by connecting to a national or international network of HPC facilities via a user-friendly web-based interface.

The CBRAIN platform provides many Neuroinformatics tools and methods including those developed by the MNI-based McGill Center for Integrative Neuroscience (MCIN) headed by Dr. Alan Evans for the study of the different types of anatomical and functional MRI techniques such as CIVET image processing (http://www.bic.mni.mcgill.ca/ServicesSoftware/CIVET-2-1-0-Introduction, MacDonald et al., 2000) and the MINC toolkit (https://github.com/BIC-MNI/minc-toolkit-v2). The platform also provides other neuroimaging pipelines from third parties such as Freesurfer (https://surfer.nmr.mgh.harvard.edu/), FSL (Smith et al., 2004; https://www.fmrib.ox.ac.uk/fsl), SPM (https://www.fil.ion.ucl.ac.uk/spm/; Friston, 2007), and others. Deployment of pipelines throughout the CBRAIN computational ecosystem automated through the use of the Boutiques JSON standard and Singularity containers that allow for machine-independent execution with no additional software development (Glatard et al., 2018). Once deployed, any user from any geographical region may benefit of the remote resources offered within the CBRAIN ecosystem. The CBRAIN computational ecosystem is comprised of multiple compute and storage resources located in Canada and around the world. At the time of writing this report, CBRAIN has a current user base of over 600 users over 191 sites in 29 countries and provides over 50 preconfigured tools for neuroscience and other research domains. This platform has let to over 60 peer-reviewed publications and has served over 100 million CPU hours of computing and 100 TBs of data creating a collaborative research network spanning the globe.

Traditionally, researchers are left to work with laborious scripting and command line interfaces to run advanced analyses on HPC resources, requiring extensive training and expertise to accomplish science. Additionally, creating large sets of experiments and aggregation and visualizing results are usually done by hand or by customized software packages. CBRAIN solves this problem by giving researchers a central location and an easy-to-use interface for submitting complicated software packages on computational resources, handling the logistics of such large-scale work behind the scenes so that scientists can concentrate on getting science done. After logging into the platform, users can utilize CBRAIN's web-based portal to upload and move data, set up and execute computational tasks, and visualize and download results on any of the high-performance computing and cloud resources registered in CBRAIN. An open-source codebase and extensive documentation for administrators enable new cloud resources or data centers to be connected to CBRAIN in a clearly documented and secure manner.

Users can have fine-grained permission control over all resources, data and tools, enabling relevant and secure sharing, and collaboration across geographically distant groups. New toolkits are provided through containerized pipelines (i.e., software installed on a light-weight virtual machine) so that they are highly portable and reproducible. Leveraging this platform for deployment enables wide access to an easily executable and live environment for the of the qEEGt toolbox, with security and cloud connectivity for user-specific datasets.

The pipelines mounted on CBRAIN for data processing and analysis facilitate the reproducibility of research and support the transparency of provenance, i.e., documenting steps to reach the same results in future and how to process other datasets. All these concepts are in line with the goals of open science.

CBRAIN is linked to the Longitudinal Online Research and Imaging System (LORIS), which is an open-source, web-based, data, and project management software aimed at storing behavioral, clinical, neuroimaging, and genetics data. LORIS is designed to gather longitudinal data from patients and to facilitate its curation and further processing. It also offers visualization tools and allows users to leverage external tools. Features include project management and study design; data collection supporting multiple modalities; workflows for data management and quality control; 3D visualization tools; and data querying and sharing tools. LORIS currently has over 400 international projects and partner sites (Das et al., 2016).

At present, CBRAIN and LORIS have developed the capability to accommodate EEG data in a standardized format in LORIS for further processing in CBRAIN. Additionally, our new pipeline has been added to CBRAIN to perform Tomographic Quantitative EEG analysis (qEEGt) of data stored either in LORIS or loaded directly via CBRAIN-connected servers. The EEG data is stored in LORIS in the newly defined BIDS-EEG format (Madjar et al., 2018; Pernet et al., 2018) to address the challenges of data exchange across projects.

## The qEEGt Plugin for CBRAIN

### User Options

We provide tools in CBRAIN capable of running quantitative analysis of EEG both at the sensors space (qEEG) and the sources level (qEEGt).

The qEEGt tool assumes that the analysis windows (epochs) have been previously selected by an expert neurophysiologist using some other system and any necessary preprocessing steps have already been performed. The selected EEG epochs are passed to the plugin and the following options are available:

a. Changing the EEG reference to any of the leads included in the recording montage or re-referencing the data to the average reference;
b. Correcting the EEG by the Global Scale Factor (GSF) (Hernández et al., 1994), which is a factor to account for a high percent of variability present in the EEG related to technical details and not to neurophysiological variability, thus, this factor makes the recordings from different devices and different persons more comparable;
c. Transforming the EEG signal to the frequency domain by means of the FFT;
d. Calculating the cross-spectral matrices for the set of leads recorded at the scalp, including the power spectra for the leads for two models: the narrow band and the broad band models;
e. Calculating the coherence^1^ and phase differences between all leads in the whole frequency range;
f. Estimating the power spectra at the sources by means of solving the EEG inverse problem, using the VARETA methodology;
g. Calculating the Z-probabilistic measurements for the spectra of the currents at the sources, using the norms of the Cuban population, in a range from 5 to 87 years old; and
h. Selecting different visualization tools.
i. A step by step guide of how to proceed to run the qEEGT pipeline in CBRAIN is provided in Appendix II.

### qEEGt Visualization

Once the qEEGT has been run (following the steps shown in Appendix II), the user can select the option to visualize the results. In that case, all the different measurements calculated are loaded in a tabbed display for showing the results in the best possible way, either as 2D topographic maps or 3D tomographic images depending on the data type.

**Figure 1:**
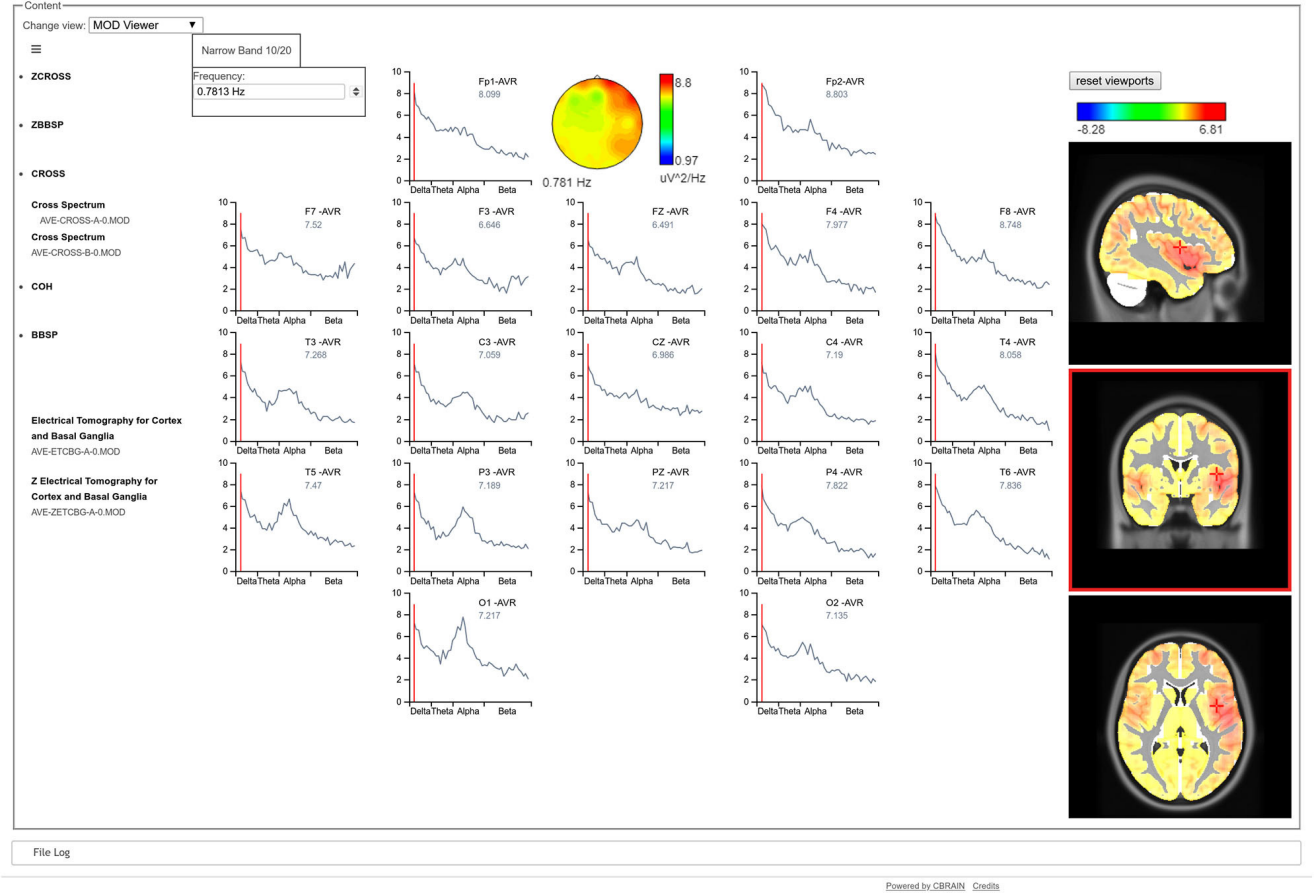
CBRAIN qEEGt visualization tool. The results of a qEEGt session are shown: the raw EEG spectra at the scalp for each electrode. A cursor indicates the specific frequency (0.78 Hz), where the spectral topographic map for all electrodes are shown. Correspondingly, the 3D tomographic view is shown for the EEG spectra at the sources for the same frequency. The red color of the topographic map at the electrodes shows an increased frontal activity, that extents to the temporal in the right hemisphere. The three views of the tomographic map show that the maximum of the activity is in the temporal pole. A similar graph can be obtained for the Z values, both at the sensors as well as at the sources.

The CBRAIN visualization tool opens a graphical user interface, in which all qEEGt results can be visualized: either raw or Z spectra at the sensors space at each frequency, synchronized with the corresponding 3D tomographic maps at the sources, for the narrow band model (high resolution spectral model) (see Figure 1).

**Figure 2:**
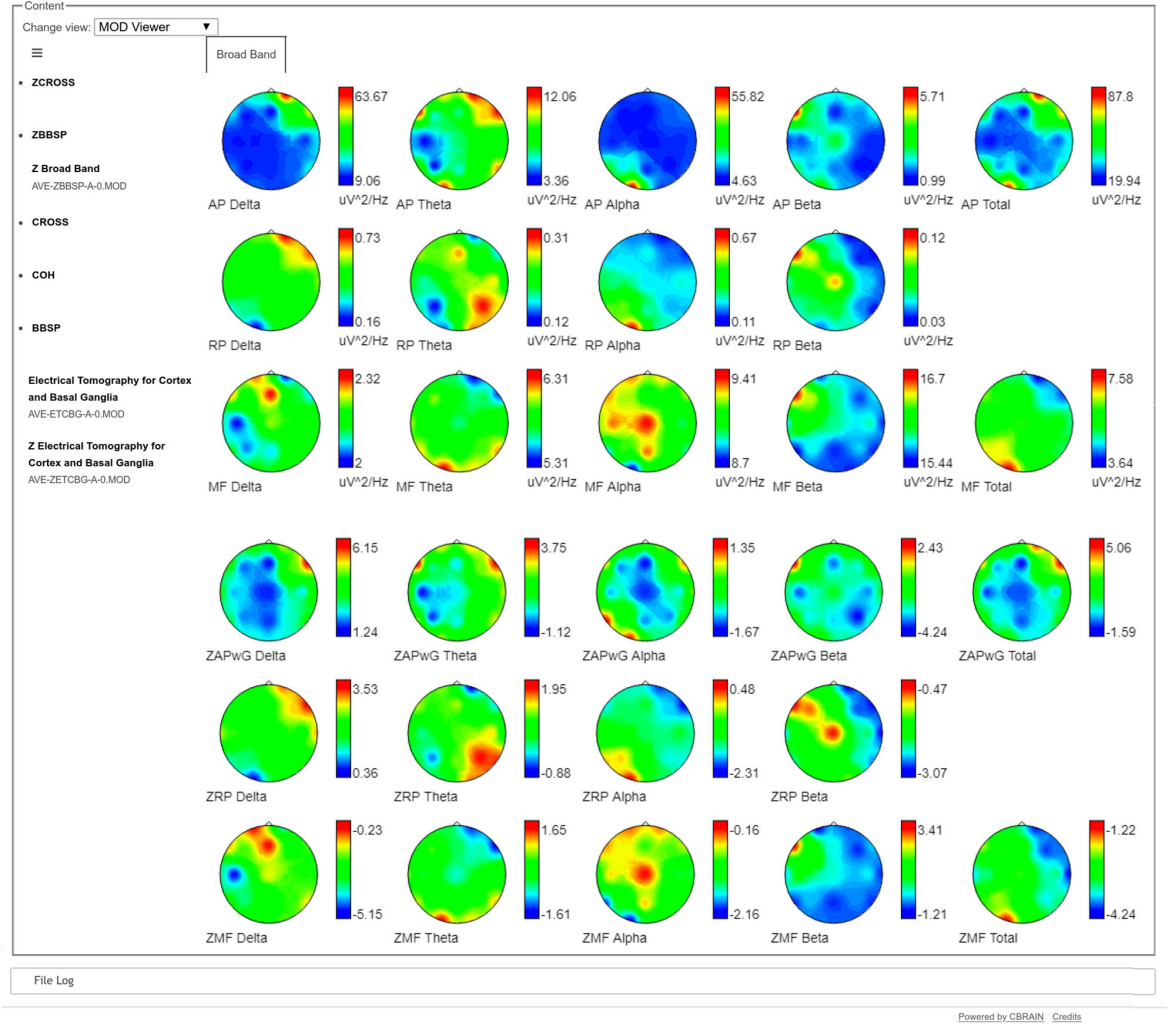
Broad Band Model visualization of raw and Z spectra. The three upper rows show the raw Absolute Power (AP), Relative Power (RP), and Mean Frequency (MF) of the individual's spectra respectively. The three bottom lines show the corresponding Z scores, calculated against the Cuban Normative Database. The red color in the raw Absolute Power maps (first row) show the same frontal and temporal higher slow activity (Delta and Theta) that was observed in Figure 1 in the right hemisphere, while the Alpha activity is concentrated in the contrary part of the contrary hemisphere (O1). The blue colors show the leads where the amplitude of the raw activity is smaller. In the case of the Z maps, the red colors indicate areas of excess of activity regarding the values of the normative database and blue colors indicate decrements of activity compared to the normative database. For example, the red colors of the Delta Z Absolute Power in the 4th row show values which are more than 6 standard deviations above the normative values. Meanwhile, in the same row, blue colors show values which are 4 standard deviations below the normative values in the parietal leads of the right hemisphere.

A compact visualization of the Broad Band Model, both for the raw spectra as well as for the normative data is also provided, as topographical maps, for the calculated frequency bands (see Figure 2). A bipolar color palette has been created for the Z-scores, which allows to highlight the negative values (decrement) in blue and positive values (excess) in red. In the case of the raw values, the same color palette is used for simplicity. Otherwise specified, the traditional bands are calculated: Delta, Theta, Beta, Alpha, Beta, and Total for the Absolute Power (AP) and the Mean Frequency (MF). Meanwhile, the Relative Power (RP) does not include the Total band.

**Figure 3:**
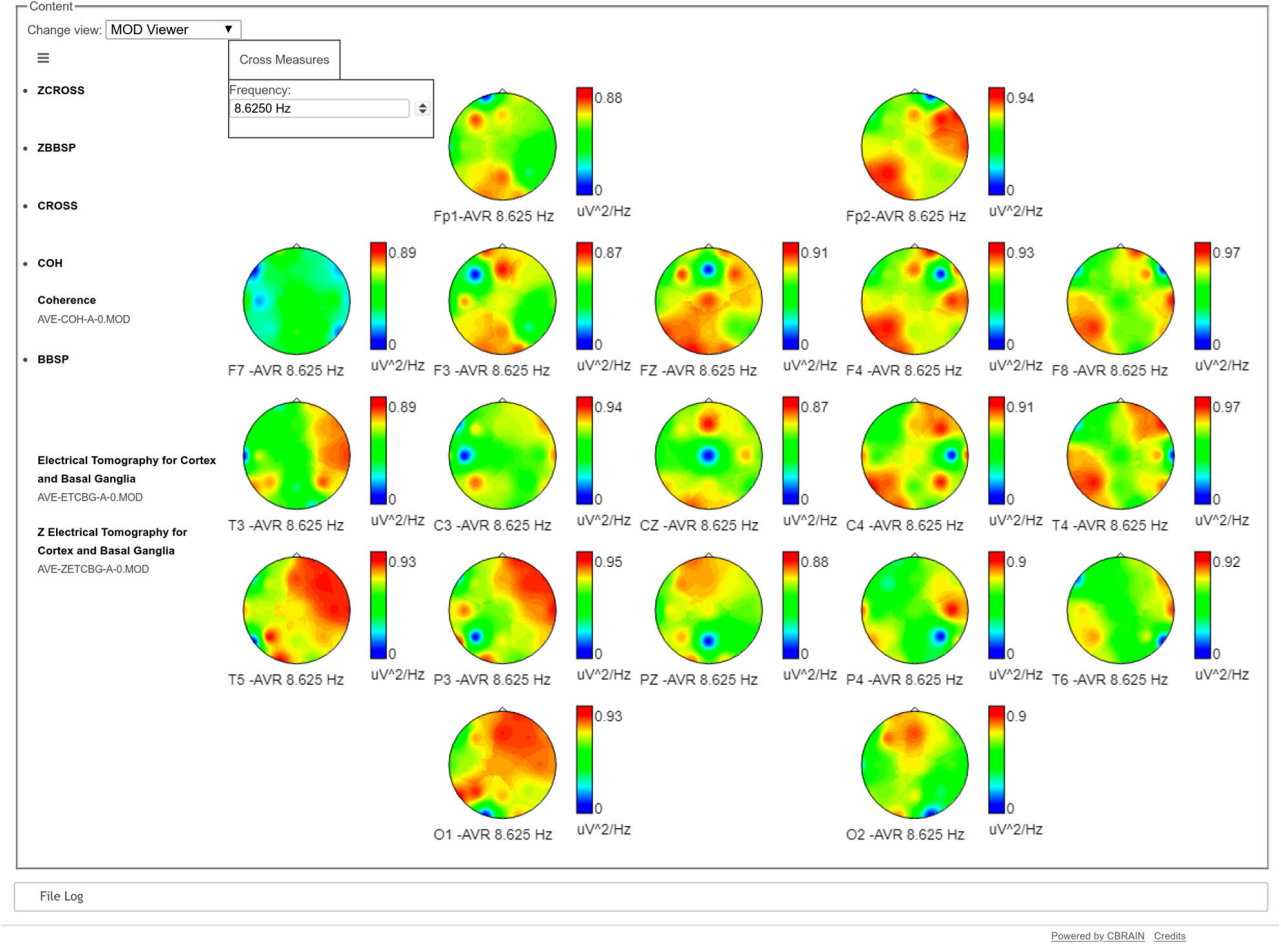
Topographical maps of the coherences of one electrode against the rest, at a specific frequency (8.6 Hz in the example). In each map, the blue dot refers to the position of the target electrode, showing its coherence regarding the rest of the head. Values in these maps go from 0 (blue) to red (1). For example, the maps of T5, P3, and O1 show a very high coherence between the parieto-occipital leads of the left hemisphere with the frontal and temporal leads of the contrary hemisphere. In the case of Fp2, it has high coherence values both with the frontal and temporal leads of its same hemisphere and the parietal and occipital leads of the contrary hemisphere.

It is also possible to visualize topographic maps of coherence, frequency by frequency, showing the coherences between one channel vs. the rest of the head, as it is shown in Figure 3.

### Example of Use: A Case Presentation

To illustrate the use of the qEEGt plugin in CBRAIN, we present the processing of an EEG study of a male patient, 71 years old, who suffered thrombotic brain stroke in the middle cerebral artery of the right hemisphere, 3 days before the EEG study. The accident produced a facial paralysis and dysarthria, visual impairment, and motor deficit in the left side of the body.

The EEG was recorded with a MEDICID IV System, sampled every 5 ms, and was edited offline. The patient was seated in a comfortable chair in a dimly lit room, with the eyes closed. The EEG was recorded from 19 leads of the 10–20 International System, using linked earlobes as a reference. A1–A2 reference was used so that the measurements were taken under the same conditions as the Cuban normative database, distributed with the qEEGt software. The amplifier bandwidth was set from 0.5 and 30 Hz. An expert electroencephalographer, visually edited the recording, selecting 24 artifact-free epochs of 2.56 s each, for the quantitative analysis.

With the qEEGt software, we first calculated the EEG spectra at the 19 electrodes of the 10/20 system, for the Eyes Closed (EC) condition. The Log of the spectra was compared against the normative EEG database of the Cuban Neuroscience Center and the probabilistic Z values were obtained for each lead and frequency.

**Figure 4:**
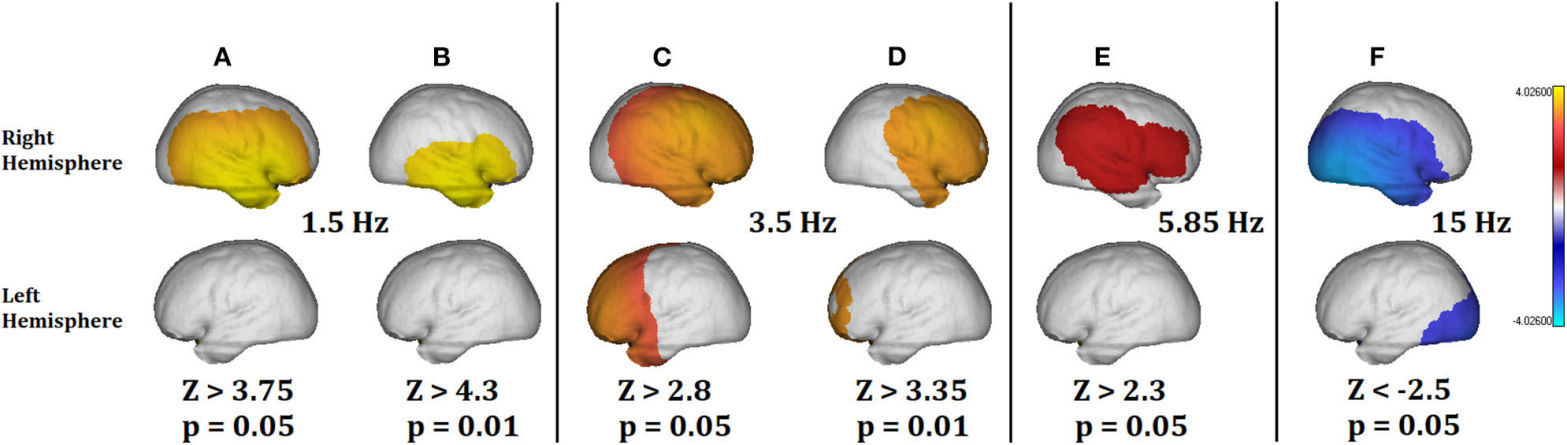
Summary of the qEEGt analysis of the 71 years old patient. The more significant results are shown for bands Delta, Theta, and Gamma at two thresholds (*p* = 0.05 and *p* = 0.01). **(A)** (*p* =0.05) and **(B)** (*p* = 0.01) show a pathological excess of Delta activity in the right hemisphere. **(C)** (*p* = 0.05) and **(D)** (*p* = 0.01) show the excess of pathological activity in the right hemisphere and frontal part of the left hemisphere. **(D)** Shows that the most pathological areas in Theta coincide with the exact location of the surrounding area of the lesion. **(E)** Shows that no pathological activity was significant at 5.85 Hz for *p* = 0.01. **(F)** Shows a pathological decrease of activity in the Beta band, related to the location of the lesion and the edema area. No significant differences were found in the Alpha band. The implications of these results are discussed in the text.

Figure 4 shows the results of the analysis for some selected frequencies: 1.5 Hz for Delta band; 3.5 and 5.85 Hz for Theta band; and 15 Hz for Beta band. The significance thresholds have been corrected for multiple comparisons using the Z maximum statistic criterion (Galan et al., 1994) for a *p* value of 0.05. However, in Theta band we show the significant areas also for *p* = 0.01 (corrected by multiple comparisons) to better highlight the more pathological areas.

Figures 4A,B show a pathological excess of Delta activity at 1.5 Hz in the right hemisphere, with a location coincident with the lesion. Figure 4B shows the pathological areas at a threshold of *p* = 0.01. The area is limited to the territory of the middle cerebral artery. At the same time, Figure 4F shows a defect of Beta activity in the same area as Figure 4A, except that it extends to the occipital area of the contrary hemisphere too.

At the limit between Delta and Theta (Figures 4C,D), the excess of pathological activity is extended to almost the whole right hemisphere and to the frontal part of the left hemisphere. The threshold at *p* = 0.01 shown in Figure 4D is to stress the point that at this frequency the most pathological areas does not coincide with the exact location of the lesion but with the surrounding area. This result is consistent with previous results by Fernández-Bouzas et al. (2002) in a qEEGt study of persons who suffered brain infarcts they found two major areas of pathological excess of slow activity: one coincident with the localization at the slower frequencies (Delta) and a second one coincident with the localization of the ischemic penumbra, which surrounds the lesion.

Note that from Figures 4A–F, the threshold value for the Z activity is decreasing with the frequency (*Z* = 3.75 at 1.5 Hz; *Z* = 2.3 at 5.85 Hz). It means that the slower the frequency the more significant pathological values. This is also consistent with the Gloor's hypothesis (Gloor et al., 1977) about the origin of the Delta waves in the brain produced by the neuronal deafferentation in the brain cortex directly below the lesion. Finally, in Figure 4E, at 5.85 Hz the pathological activity was only significant at the threshold of *p* = 0.05 but not for *p* = 0.01 after correction for multiple comparisons. In the same way, there was no significant pathological activity in Alpha band.

**Figure 5:**
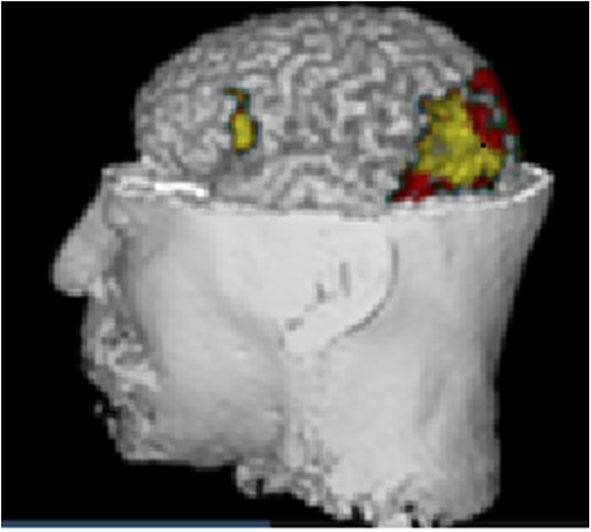
Morphometric analysis of the T1 MRI of the same case. Statistical parametric mapping using the plugin IBASPM based on the regional volume of the MRI in comparison with the normative data (Z values). The yellow area identified the regions with brain damage, occipital damage responsible of visual impairment, and lesion in Broca's area which explained the language impairment, not detected by MRI. The correspondence with Figure 4 is striking.

As an independent confirmation we show a morphometric analysis of the T1 MRI of the same person (Figure 5). The statistical parametric map was performed using the plugin IBASPM (https://www.fil.ion.ucl.ac.uk/spm/ext/#IBASPM) based on the regional volume of the MRI. The subject's values were compared with the normative values obtained from the Cuban Normative database (Z values). The yellow color identified the regions with brain damage: occipital damage responsible of visual impairment; and the lesion in Broca's area may explain the language impairment, not detected by MRI. These two abnormality patterns are in consonance with the qEEGt findings shown in Figures 4C,F.

This case presentation of a clinical patient is only to illustrate the use of the toolbox and its possibilities. This section is not intended as a validation of the VARETA methodology, which has been widely used and validated for many years in different clinical and experimental settings. A non-exhaustive list of about 200 citations retrieved from Google Scholar in September 2019 (excluding auto citations) is provided as a supplemental material (53 of them belong to the period 2013–2019).

It is also important to emphasize that in this toolbox we do not include statistical tools for group analysis of neuroimages. The z score is only an intermediate step, useful for visualization purposes and classification. The true multivariate nature of the data must be considered in further applications. Tools for group statistics of neuroimages developed at the Cuban Neuroscience Center will be added as CBRAIN plugins in the future, for example, Mahalanobis maps (Galan et al., 1994) for visualization, or stable biomarker identification (Bosch-Bayard et al., 2018) among others.

## Implementation Details and Challenges

The Matlab code of the qEEGt procedure was modified to condense it in a single procedure, which performs all the analysis and produces all the necessary outputs. This procedure was compiled and use as the input for the CBRAIN plugin.

A Boutique JSON descriptor for qEEG (doi: 10.5281/zenodo.1451003) was created to define the format of the command line execution and the various options that can be set. Then a Singularity container was created to provide a machine independent installation of the qEEG tool. Finally, CBRAIN can automatically import the Boutique descriptor to create the tool and deploy it for users.

The highly interactive qEEGt visualization capability is built in the highly modular ReactJS framework (which makes it independent of the computer platforms) with any modern internet browser. The application is designed as an SDI (Single Document Interface) with a graphical user interface oriented to provide the maximum amount of information with the minimum amount of user input.

Note that there are facilities for the creation of data structures for storing EEG information in the LORIS data platform as well as the implementation within the CBRAIN high-performance computing platform of a core of tools developed at CNEURO.

## Discussion and Conclusions

The present qEEGt plugin is the first step to introduce EEG functionalities in CBRAIN, one of the most widely used ecosystems for brain imaging analysis. Will facilitate the extended use the qEEGt method and toolbox, which has proven to be a useful tool for the quantitative EEG analysis, both at the electrode and at the sources level.

This method introduced the concept of “normative SPM of EEG source spectra” based on the use of EEG normative databases. The resulting SPM “z maps” that compare individuals to age appropriate norms is an essential pre-processing tool which facilitates assessment of pathological states (or at least deviation from normality). It is to be noted that the normative data encoded in the regression equations of the current qEEGt toolbox are the first open source information of this type. Importantly, they are not only for the usual brain states available in most databases namely Eyes Closed and/or Eyes Open, but also include other states that are clinically relevant such as Hyperventilation. Although these tools are still mainly used for research, they have a clinical usefulness, specifically the assessment of deviation from normality (Nuwer et al., 1994, 1999; Babiloni et al., 2019a).

The plugin resulted easy to operate for totally naive users in a reasonable time for a single task. Familiarity with the tool and batch processing of several EEG recordings will increase productivity with the toolbox. Nevertheless we continue to evaluate both the CBRAIN interface as well as the toolbox to increase its user friendliness.

As mentioned in the introduction, our development of qEEG was based on the Cuban Human Brain Mapping Project (CHBMP), which was carried out in three waves: the first one (1988–2003) acquired the EEG of 211 subjects aged 5–87, randomly selected from the general population. At that time, only EEG was recorded since there were no MRI systems available. While useful in setting for which MRI is not feasible or costly, future work must extend the methods to individualized brain morphology. This is the more necessary since though there has been work with EEG SPM in individualized source space (Park et al., 2002), it yet has to be extended to age corrected normative measures.

Fortunately two subsequent waves (2004–2014) and (since 2018) of the CHBMP have been launched since then, which now included individualized MRI, DWI as well as high resolution EEG (more details in Hernandez-Gonzalez et al., 2011). These projects have generated normative data that has been used in different Public Health Systems (Hernandez-Gonzalez et al., 2011; Valdés-Sosa et al., 2018) which will lead to successive versions of qEEGt in CBRAIN, and whose results will be described in further publications and will certainly address individualized head models.

Among the additional facilities to be included to qEEGt soon are:

d) Individualized head geometry for Human Connectome compatible pipelines
e) Improved approximate head models (Valdés-Hernández et al., 2009) for situations in which MRI is difficult to acquire.
f) Extension of EEG sources features to non-stationary and nonlinear phenomena.
g) Inclusion of third generation methods for joint estimation of EEG source activation and connectivity (Paz-Linares et al., 2017, 2018).
h) A toolbox for biomarker selection from EEG source features (Bosch-Bayard et al., 2018).

This step, of making our toolbox available in CBRAIN (as well as the methods in Github) will allow us to place in the public scrutiny the procedures and, we hope, to increase the contributions and interactions with other similar efforts.

## Data Availability Statement

All datasets generated for this study are included in the article/Supplementary Material.

## Author Contributions

PV-S contributed key concepts of the qEEG, the mathematical definition of VARETA and qEEGt VARETA, wrote and revised the paper, and directed and organized the three waves of the Cuban Human Brain Mapping projects (CHBMP). JB-B programmed all the algorithms, created the Norms, methods testing, wrote the paper, and participated in dockerization in CBRAIN. EA-V created all visualization tools and the user-friendly version of the qEEGT software. SB organized and directed the qEEGt dockerization in CBRAIN and throughout paper revision. CR participated in qEEGt integration with LORIS and throughout paper revision. GK, TG, and LS carried out the qEEGt dockerization in CBRAIN and wrote the qEEGT web interface for CBRAIN. LG-G developed all the statistical methods of qEEGT as well as permutations methods for high dimensional datasets. MB-V and TV-A participated in the clinical evaluation of qEEGt. AT wrote the web visualization for the qEEGT in CBRAIN. SD, CM, ZM, and LM participated in qEEGt integration in LORIS, discussions, and paper revision. Evans organized, facilitated, supervised, and provided the environment and resources for qEEGt integration in CBRAIN, as well as paper revision. JB-B, EA-V, LG-G, and TV-A participated and carried out in the three waves of CHBMP and have worked in the topic for more than 25 years. All authors contributed to the article and approved the submitted version.

## Conflict of Interest

The authors declare that the research was conducted in the absence of any commercial or financial relationships that could be construed as a potential conflict of interest.

## Footnotes

^1^The coherence matrix has been included in this toolbox because of historical reasons, although this is not a connectivity toolbox. The coherence is a basic measurement that is included in most of the quantitative analysis. This measurement is widely used in EEG research to assess electrical symmetry between pairs of homologues channels (right-left hemispheres same position channels) rather than for connectivity. This use is important to assess normal brain functioning and it is of clinical importance. It is not intended to be a measurement of connectivity. It is a well-known fact that any connectivity measurement at the scalp is affected by the volume conduction effect. Therefore, none of the recently defined methods to estimate brain coupling in the frequency domain can provide a real estimation of the functional connections among the brain regions. Additionally, Nolte et al. (2019) have shown that most of the phase coupling measurement are in fact a function of the complex coherency.

## References

[B1] Babiloni C. Barry R. J. Başar E. Blinowska K. J. Cichocki A. Drinkenburg W. H. I. M.. (2019a). International Federation of Clinical Neurophysiology (IFCN)—EEG research workgroup: recommendations on frequency and topographic analysis of resting state EEG rhythms. Part 1: applications in clinical research studies. Clin. Neurophysiol. 131, 285–307. 10.1016/j.clinph.2019.06.234 31501011

[B2] Babiloni C. Del Percio C. Pascarelli M. T. Lizio R. Noce G. Lopez S.. (2019b). Abnormalities of functional cortical source connectivity of resting-state electroencephalographic alpha rhythms are similar in patients with mild cognitive impairment due to Alzheimer's and Lewy body diseases. Neurobiol. Aging 77, 112–127. 10.1016/j.neurobiolaging.2019.01.013 30797169

[B3] Bosch-Bayard J Valdés-Sosa P. Virues-Alba T. Aubert-Vázquez E. John E. R. Harmony T.. (2001). 3D statistical parametric mapping of EEG source spectra by means of variable resolution electromagnetic tomography (VARETA). Clin. EEG 32, 47–61. 10.1177/155005940103200203 11360721

[B4] Bosch-Bayard J. Galán-García L. Fernandez T. Lirio R. B. Bringas-Vega M. L. Roca-Stappung M.. (2018). Stable sparse classifiers identify qEEG signatures that predict learning disabilities (NOS) severity. Front. Neurosci. 11:749. 10.3389/fnins.2017.00749 29379411 PMC5775224

[B5] Bosch-Bayard J. Valdés-Sosa P. A. Fernandez T. Otero G. Pliego B. Ricardo-Garcell J.. (2012). NeuroImage 3D statistical parametric mapping of quiet sleep EEG in the first year of life. NeuroImage 59, 3297–3308. 10.1016/j.neuroimage.2011.11.001 22100773

[B6] Das S. Glatard T. MacIntyre L. C. Madjar C. Rogers C. Rousseau M.-E.. (2016). The MNI data-sharing and processing ecosystem. NeuroImage 124(Pt B), 1188–1195. 10.1016/j.neuroimage.2015.08.076 26364860

[B7] Das S. Zijdenbos A. P. Harlap J. Vins D. Evans A. C. (2012). LORIS: a web-based data management system for multi-center studies. Front. Neuroinf. 5:37. 10.3389/fninf.2011.00037 22319489 PMC3262165

[B8] Evans A. Collins D. Millst S. Brown E. Kelly R. Peters T. (1993). 3D statistical neuroanatomical models from 305 MRI volumes, in Proceedings of IEEE—Nuclear Science Symposium and Medical Imaging Conference (San Francisco, CA), 1813–1817. 10.1109/NSSMIC.1993.373602

[B9] Farzan F. Atluri S. Frehlich M. Dhami P. Kleffner K. Price R.. (2017). Standardization of electroencephalography for multi-site, multi-platform and multi-investigator studies: insights from the canadian biomarker integration network in depression. Sci. Rep. 7:7473. 10.1038/s41598-017-07613-x 28785082 PMC5547036

[B10] Fernández-Bouzas A. Harmony T. Fernández T. Aubert E. Ricardo-Garcell J. Valdés P.. (2002). Sources of abnormal EEG activity in spontaneous intracerebral hemorrhage. Clin. EEG 33, 70–76. 10.1177/155005940203300205 12025734

[B11] Fernández-Bouzas A. Harmony T. Galán L. Marosi E. Fernández T. Reyes A.. (1995). Comparison of Z and multivariate statistical brain electromagnetic maps for the localization of brain lesions. Electroencephal. Clin. Neurophys. 95, 372–380. 10.1016/0013-4694(95)00111-B 7489666

[B12] Friston K. (2007). Statistical parametric mapping in Statistical Parametric Mapping: The Analysis of Funtional Brain Images, 1st Edn, eds W. Penny, K. Friston, J. Ashburner, S. Kiebel, and T. Nichol (Amsterdam; Boston, MA: Elsevier/Academic Press).

[B13] Friston K. J. Holmes A. P. Worsley K. J. Poline J. B. Frith C. D. Frackowiak R. S. J. (1995). Statistical parametric maps in functional imaging: a general linear approach. Hum. Brain Mapp. 2, 189–210. 10.1002/hbm.46002040217383900

[B14] Galan L. Biscay R. Valdes P. Neira L. Virues T. (1994). Multivariate statistical brain electromagnetic mapping. Brain Topogr. 7, 17–28. 10.1007/BF01184834 7803196

[B15] Glatard T. Kiar G. Aumentado-Armstrong T. Beck N. Bellec P. Bernard R.. (2018). Boutiques: a flexible framework to integrate command-line applications in computing platforms. GigaScience 7:giy016. 10.1093/gigascience/giy016 29718199 PMC6007562

[B16] Gloor P. Ball G. Schaul N. (1977). Brain lesions that produce delta waves in the EEG. Neurology 27, 326–333. 10.1212/WNL.27.4.326 557774

[B17] Hernández J. L. Valdés P. Biscay R. Virues T. Szava S. Bosch J.. (1994). A global scale factor in brain topography. Int. J. Neurosci. 76, 267–278. 10.3109/00207459408986009 7960483

[B18] Hernandez-Gonzalez G. Bringas-Vega M. L. Galán-García L. Bosch-Bayard J. Melie-Garcia Y. L. L. Valdes-Urrutia L.. (2011). Multimodal quantitative neuroimaging databases and methods: the cuban human brain mapping project. Clin. EEG Neurosci. 42, 149–159. 10.1177/155005941104200303 21870466

[B19] John E. Karmel B. Corning W. Easton P. Brown D. Ahn H.. (1977). Neurometrics. Science 196, 1393–1410. 10.1126/science.867036 867036

[B20] MacDonald D. Kabani N. Avis D. Evans A. C. (2000). Automated 3-D extraction of inner and outer surfaces of cerebral cortex from MRI. NeuroImage 12, 340–356. 10.1006/nimg.1999.0534 10944416

[B21] Madjar C. Rogers C. Desjardins J. Lo D. Safi-Harab M. Legault M. (2018). Electrophysiology data sharing: inserting standardized EEG data into the LORIS framework using BIDS, in INCF Congress: Neuroinformatics 2018, ed G. Node (Montreal, QC), 90.

[B22] Michel C. M. Murray M. M. Lantz G. Gonzalez S. Spinelli L. Grave De Peralta R. (2004). EEG source imaging. Clin. Neurophysiol. 115, 2195–2222. 10.1016/j.clinph.2004.06.001 15351361

[B23] Niedermeyer E. Schomer D. L. Lopes da Silva F. H. (2010). Niedermeyer's Electroencephalography leBasic Principles, Clinical Applications, and Related Fields. Philadelphia, PA; Baltimore, MD: Wolters Kluwer Health 10.1093/med/9780190228484.001.0001

[B24] Nolte G. Galindo-Leon E. Li Z. Liu X. Engel A. K. (2019). Mathematical relations between measures of brain connectivity estimated from electrophysiological recordings for Gaussian distributed data. bioRxiv 680678. 10.1101/680678 PMC7683718 33240037

[B25] Nunez P. L. Nunez M. D. Srinivasan R. (2019). Multi-scale neural sources of EEG: genuine, equivalent, and representative. A tutorial review. Brain Topogr. 32, 193–214. 10.1007/s10548-019-00701-3 30684161

[B26] Nuwer M. R Lehmann D. da Silva F. L. Matsuoka S. Sutherling W. Vibert J. F. (1999). IFCN guidelines for topographic and frequency analysis of EEGs and EPs. The International Federation of Clinical Neurophysiology. Electroencephalogr. Clin. Neurophysiol. Suppl. 52, 15–20. 10590973

[B27] Nuwer M. R. Lehmann D. Lopes da Silva F. Matsuoka S. Sutherling W. Vibert J.-F. (1994). IFCN guidelines for topographic and frequency analysis of EEGs and EPs. Report of an IFCN committee. Electroencephalogr. Clin. Neurophysiol. 91, 1–5. 10.1016/0013-4694(94)90011-6 7517838

[B28] Pardoux E. (2008). Markov Processes and Applications: Algorithms, Networks, Genome and Finance. West Sussex: Wiley 10.1002/9780470721872

[B29] Park H.-J. Kwon J. S. Youn T. Pae J. S. Kim J.-J. Kim M.-S.. (2002). Statistical parametric mapping of LORETA using high density EEG and individual MRI: application to mismatch negativities in Schizophrenia. Hum. Brain Mapp. 17, 168–178. 10.1002/hbm.10059 12391570 PMC6872044

[B30] Paz-Linares D. Gonzalez-Moreira E. Bosch-Bayard J. Areces-Gonzalez A. Bringas-Vega M. L. Valdes-Sosa P. A. (2018). Neural connectivity with hidden gaussian graphical state-model. arXiv[Preprint]. *arXiv:1810.01174*. Available online at: https://arxiv.org/abs/1810.01174v3

[B31] Paz-Linares D. Vega-Hernández M. Rojas-López P. A. Valdés-Hernández P. A. Martínez-Montes E. Valdés-Sosa P. A. (2017). Spatio temporal EEG source imaging with the hierarchical Bayesian elastic net and elitist lasso Models. Front. Neurosci. 11:635. 10.3389/fnins.2017.00635 29200994 PMC5696363

[B32] Pernet D. C. Appelhoff S. Flandin G. Phillips C. Delorme A. Oostenveld R. (2018). BIDS-EEG: an extension to the brain imaging data structure (BIDS) specification for electroencephalography. PsyArxiv. 10.31234/osf.io/63a4y 31239435 PMC6592877

[B33] Sherif T. Rioux P. Rousseau M.-E. Kassis N. Beck N. Adalat R.. (2014). CBRAIN: a web-based, distributed computing platform for collaborative neuroimaging research. Front. Neuroinf. 8:54. 10.3389/fninf.2014.00054 24904400 PMC4033081

[B34] Smith S. M. Jenkinson M. Woolrich M. W. Beckmann C. F. Behrens T. E. J. Johansen-Berg H.. (2004). Advances in functional and structural MR image analysis and implementation as FSL. NeuroImage 23, S208–S219. 10.1016/j.neuroimage.2004.07.051 15501092

[B35] Szava S. Valdes P. Biscay R. Galan L. Bosch J. Jimenez J. C. (1994). High resolution quantitative EEG analysis. Brain 6, 211–219. 10.1007/BF01187711 8204408

[B36] Valdés-Hernández P. A. von Ellenrieder N. Ojeda-Gonzalez A. Kochen S. Alemán-Gómez Y. Muravchik C. Valdés-Sosa P. A. (2009). Approximate average head models for EEG source imaging. J. Neurosci. Methods 185, 125–132. 10.1016/j.jneumeth.2009.09.005 19747944

[B37] Valdés-Sosa P. A. Galler J. R. Bryce C. P. Rabinowitz A. G. Bringas-Vega M. L. Hernández-Mesa N.. (2018). Seeking biomarkers of early childhood malnutrition's long-term Effects. MEDICC Rev. 20, 43–48. 10.37757/mr2018.v20.n2.10 29773777 PMC6310420

[B38] Vega-Hernández M. Martínez-Montes E. Sánchez-Bornot J. M. Lage-Castellanos A. Valdés-Sosa P. A. (2008). Penalized least squares methods for solving the EEG inverse problem. Stat. Sin. 18, 1535–1551. Available online at: https://www.jstor.org/stable/24308568

[B39] Wang Y. Van de Moortele P.-F. He B. (2019). Automated gradient-based electrical properties tomography in the human brain using 7 Tesla MRI. Magn. Reson. Imaging 63, 258–266. 10.1016/j.mri.2019.08.003 31425805 PMC6861698

