# Supplementary material for: A Quantitative EEG Toolbox for the MNI Neuroinformatics Ecosystem: Normative SPM of EEG Source Spectra
Source: Front Neuroinform. 2020 Aug 7;14:33. doi: 10.3389/fninf.2020.00033 (PMC7427620; doi:10.3389/fninf.2020.00033)
Supplement: Supplementary file 1 [file Data_Sheet_1.pdf]

Appendix I: qEEGT-VARETA

For easier reference here follows a summary of the Variable Resolution Electromagnetic Tomography (VARETA) methodology in the frequency domain (Bosch-Bayard et al., 2001):

Variable Resolution Electrical tomography with the MNI average brain.

For analysis of the EEG background activity in clinical practice, we recommend the selection of EEG epochs between 2- and 2.56-seconds of length, after artifact rejection and guaranteeing the signal to be quasi-stationary. Approximately 24 EEG epochs should be selected. This in accordance with the IFCN guidelines (Babiloni et al., 2019).

In accordance with this let $\mathbf{v}_{i,j}^{raw}(t) \in \Re^{Nd}$ denote the vector of raw EEG signal recorded at the scalp, for the Nd leads at time t. The sub index $i=1...Ns$, denotes subjects, $j=1...Ne$ (typically 24) is the number of epochs, and $t=1...Nt$ (typically 512) is the number of time instants in each epoch.

The raw signal is transformed to the average reference:

(1) $$\mathbf{v}_{i,j}(t) = \mathbf{H}\,\mathbf{v}_{i,j}^{raw}(t)$$

Here $\mathbf{H} = \mathbf{I}_{Nd} - \mathbf{1}_{Nd \times Nd}$ is the centering matrix (Mardia, Kent, & Bibby, 1997, pp. 270).

To enable a tomographic analysis which describes the spectral sources at the different frequencies without the need of considering interactions, it is desirable to construct a description in which frequencies are independent. Under this assumption, transformation to the frequency domain by the Fast Fourier Transform (FFT) (yielding a complex Gaussian distribution) is equivalent to a Principal Components Analysis which, in the presence of a Gaussian distribution, provides components that are independent by definition. Of course, for more general distributions the vanishing of higher order moments is also required but in this case the limit theorem of Brillinger (Brillinger, 1974) guarantees the validity of our assumptions which have been further ensured checking with a multivariate box cox transformation (Biscay Lirio et al., 1989).

$\mathbf{v}_{i,j}(t)$ is then transformed to the frequency domain by means of the FFT producing a set of complex vectors $\mathbf{v}_{i,j}(\omega)$, where $\omega = 1...N\omega$ denotes the frequency index. With the EEG sampling specifications given above, N_{ω}=49 (number of frequencies), for a frequency span from 0.39 Hz to 19.11 Hz with frequency bins of 0.39 Hz. This is the bandwidth presently used in the Cuban Normative database for the qEEGt (the VARETA Statistical Parametric Mapping).

In the context of the assumptions specified above, the central limit theorem (Brillinger, 1974) guarantees that $\boldsymbol{V}_{ij}(\omega)$ is distributed as a Complex Multivariate Gaussian vector which is independent from all vectors at all other frequencies. This vector has zero mean and is used to calculate a complex hermitian covariance matrix $\Sigma_i(\omega)$ which is estimated by:

(2) $$S_i^u(\omega) = \frac{1}{Ne}\sum_{j=1}^{Ne} \mathbf{v}_{i,j}(\omega).\mathbf{v}_{i,j}(\omega)^*$$

where * denotes the conjugate transpose of a vector and the u superscript stands for "uncorrected".

The correction by the Global Scale Factor (GSF) (Hernández et al., 1994) is achieved by the following expression:

(3) $$S_i(\omega) = \frac{1}{\gamma_i^2} S_i^u(\omega)$$

(Hernández et al., 1994; Valdés et al., 1992) have shown that EEGs differ greatly by a random scale factor that affects equally all channels, which is subject and recording specific. Hernandez et al showed that EEGs differ greatly by a random scale factor that affects equally all channels. This is named the Global Scale factor. This has been shown to useful also to compensate for slight differences in amplifier systems since the norms are all Cuban. This correction consists of a subtracting from the log spectra of each electrode and frequency the overall average:

(4) $$\gamma_i = \exp\left[\frac{1}{N_d . N_\omega} \sum_{d=1}^{Nd} \sum_{\omega=1}^{N\omega} \log\left(S_i^{dd}(\omega)\right)\right]$$

where $S_i^{lm}(\omega)$ is the ***(l, m)*** component of the cross spectral matrix for subject i; ***exp*** is the exponential function and log is the natural logarithm. $S_i^{dd}(\omega)$ is the power spectrum at frequency $\boldsymbol{\omega}$. This has been shown to be useful also to compensate for slight differences in amplifier systems since the norms are all from Cuba.

Frequency domain VARETA can be specified as the estimation of $\mathbf{j}$ from the following forward problem (Bosch-Bayard et al., 2001):

(5) $$\mathbf{v}_{ir}(\omega) = \mathbf{K}\,\mathbf{j}_{ir}(\omega) + \mathbf{e}_{ir}(\omega)$$

where $\mathbf{v}_{ir}(\omega)$ denotes the true data and $\mathbf{e}_{ir}(\omega)$ refers to error contributions from influences such as impedance fluctuations on the sensor array. $\mathbf{j}_{ir}(\omega)$ is the matrix of the (X, Y, Z) components of the primary current field discretized on a grid inside the gray matter of the brain (3244 grid points in the current implementation). The operator $\mathbf{K}$ is the lead field matrix that relates the current densities with the observed EEG voltage, which is obtained by spatial discretization (Riera, Aubert, Valdés, Casanova, & Lins, 1996). The Lead field has also been transformed to the average reference, according to Pascual-Marqui (2007). In this version of VARETA, the grid is defined over the MRI Atlas of the Montreal Neurological Institute (MNI) (Evans et al., 1993).

The VARETA inverse solution (Valdes-Sosa, Garcia, & Casanova, 1996) for this problem is obtained by minimizing the following objective function:

$$\sum_{r=1}^{Ne}\left(\left(v_{ir}(w)\text{-}K.j_{ir}(w)\right)^{t}.S_{Ei}^{-1}(w).\left(v_{ir}(w)\text{-}K.j_{ir}(w)\right)+j_{ir}(w)^{t}.S_{Ji}^{-1}(w).j_{ir}(w)\right)+ \quad (6)$$
$$(m+Ne).\ln\left|S_{Ji}(w)\right|+\frac{1}{t_{i}^{2}(w)}Tr\left\{S_{Ji}^{-1}(w).G\right\}$$

where τ is the regularization parameter and $\mathbf{G}=\left(\Lambda_{S}\bullet L_{3}^{t}\bullet\Lambda_{m}^{2}\bullet L_{3}\bullet\Lambda_{S}\right)^{-1}$

This is a hierarchical generalization of the usual Bayesian formulation for inverse problems (Tarantola, 1987). It should be noted that to minimize the expression (6) $\mathbf{j}_{ir}(\omega)$ must be the outcome of a trade-off between several factors:

1. $\sum_{r=1}^{Ne}\left(\left(\mathbf{v}_{ir}(\omega)-K.J_{ir}(\omega)\right)^{t}.\Sigma_{Ei}^{-1}(\omega).\left(\mathbf{v}_{ir}(\omega)-K.J_{ir}(\omega)\right)\right)$ is the term that expresses the usual goodness of fit between the data and the model. $\Sigma_{\boldsymbol{E}_i}$ is the noise covariance matrix.
2. $\sum_{r=1}^{Ne}\left(J_{ir}(\omega)^{t}.S_{Ji}^{-1}(\omega).J_{ir}(\omega)\right)$ imposes the mask upon the solution, which defines those voxels in which sources of the EEG are to be permitted. These assumptions are contained in the source covariance matrices $S_{\mathbf{J}_i}(\omega)$.
3. The rest of the terms result from placing a natural conjugate prior on $\sum_{Ji}(\omega)$ (Mardia et al., 1997, pp 109-111) in which the a priori covariance matrix is proportional to $\boldsymbol{G}$.

Matrix $\boldsymbol{G}$ incorporates several assumptions about the sources which defines the *regularization parameter*:

a. the spatial smoothness is determined by $\boldsymbol{L}_3$, which is the Kronecker product of $\boldsymbol{L}$ with the Identity matrix $\mathbf{I_3}$. $\boldsymbol{L}$ is any scalar roughness operator such as the Laplacian or thin plate spline operator. In this paper, the Laplacian operator is adopted as in (R D Pascual-Marqui, Michel, & Lehmann, 1994) and (Riera et al., 1996), in which it is introduced for the first time a numerical approach for calculating the EEG inverse solutions at the cortical surface.

b. Λ_S is a diagonal matrix which specifies the amount of smoothness at every source in the gray matter. This is an important difference with LORETA, which considers that the spatial smoothness in all sources is the same. This parameter is known as the regularization parameter.

c. Matrix Λ_g is a diagonal matrix that defines the a priori probability for each source to produce current. Large values of smoothing force constant solutions. Zero values specify no smoothing, i.e., point-solutions.

d. Λ_m is a diagonal matrix that defines the a priori probability obtained from the mask, that there might be any primary current density at a given location.

In this work $\Lambda_m = \mathbf{W}.\Lambda_G^{\frac{1}{2}}$, where **W** is the weights matrix. It is a diagonal weights matrix**,** introduced by (George et al., 1995) to attenuate the high bias of the inverse solutions to the superficial sources. The diagonal matrix Λ_G contains an estimate of the probability that there may be cortical gray matter for each point in the solution grid. These probabilities are available from the MNI probabilistic tissue maps from the MNI.

In its more general form, the estimation of VARETA is performed by the Expectation-Maximization (EM) algorithm (Dempster, Laird, & Rubin, 1977) in which $\tau_i(\omega)$ is estimated by minimizing the Generalized Cross-validation Criterion (GCV) (Casanova et al., 2000). The EM-algorithm usually converges only to local minima and, in the general case slows down near the optimal point. However, after extensive examination of the dataset acceptable starting points for any EEG were found, including a restricted range of regularization parameters. Essentially, an estimate of the source spectra is obtained for each voxel and frequency by interpolation from the neighboring voxels and compared with the values allocated to that voxel. This nonlinear estimation procedure may achieve super resolution and eliminate "ghost solutions" that are artifacts of simple linear inverse solutions.

From the previous section, an estimate of the currents at the sources $J_i(\omega)$ for each frequency can be obtained. Topographic methods in the frequency domain are based upon estimates of the source cross-spectral matrices $S_{J_i}(\omega)$. An algorithm simplification was introduced to allow the efficient estimation of this parameter. That simplification consists in substituting the data set $J_i(\omega) = \left[J_{i,1}(\omega), J_{i,2}(\omega), \ldots, J_{i,Ne}(\omega) \right]$ at a given frequency ω by its statistically equivalent expression $\boldsymbol{S}_i^{\frac{1}{2}}(\omega)$, which is the square root of its "ideal" symmetric cross spectral matrix at the EEG channels.

$S_i^{\frac{1}{2}}(\omega) = \psi_i(\omega).diag\left(\lambda_{i,j}^{\frac{1}{2}}(\omega) \right).\psi_i^*(\omega)$, where $\psi_i(\omega)$ is the eigenvectors matrix and $diag\left(\lambda_{i,j}^{\frac{1}{2}}(\omega) \right)$ is a diagonal matrix which contains the square root of the eigenvectors. $S_i(\omega)$ is the estimation of the cross spectral matrices at the sources is an iterative procedure, which is described as follows:

Starting: The matrix $\Sigma_i(\omega)$ is initialized with the value:

(7) $\boldsymbol{\Sigma}_{\mathbf{J}_i}^0(\omega) = \Lambda_G \cdot \mathbf{W} \cdot L_3^t \cdot \Lambda_G^2 \cdot L_3 \cdot \mathbf{W} \cdot \Lambda_G$

k-th iteration: The following terms are calculated:

$$A_{J_i}(\omega)^{(k)} = \Sigma_{J_i}(\omega)^{(k-1)} \cdot \mathbf{K}^t \cdot \left(\mathbf{K} \cdot \Sigma_{J_i}(\omega)^{(k-1)} \cdot \mathbf{K}^t + \Sigma_{Ei}(\omega)\right)^{-1} \cdot S_i^{\frac{1}{2}}(\omega)$$

(8) $$\Sigma_{J_i}(\omega)^{(k)} = \frac{Ne.A_{J_i}(\omega)^{(k)} \cdot A^*_{J_i}(\omega)^{(k)} + \frac{1}{\tau_i^2(\omega)}\mathbf{G}}{m + Ne}$$

The process is repeated until the estimates converge.

An important point to emphasize is that in expressions (7) and (8) it is necessary to use the full cross-spectral matrices of the data in order to obtain an estimate of the source cross-spectrum. It is therefore incorrect to attempt to fit sources by means of just using the power spectra of the EEG data.

Simplifications to speed-up the calculations

The procedure above described can be time consuming. To speed up the calculations, important simplifications are introduced:

a) Λ_G is simplified to a mask of 0s and 1s by thresholding the probability mask. In this work we use a threshold of 0.4, which is imposed to the probability mask of the MNI Atlas. A value of zero indicates not belonging to the gray matter and a value of 1 indicates the source as gray matter.

b) Λ_S is taken a priori equal to Λ_G

c) the hyperparameter $\tau_i(\omega)$ is fixed a priori to a small value, calculated as the mean of the hyperparameters obtained with GCV from all 211 subjects of the normative database, with careful inspection of each solution. This simplification makes the inter subjects' solutions comparable, allowing for further statistical comparisons.

d) The estimation procedure is stopped after the first iteration.

e) It is assumed that $\Sigma_{\boldsymbol{E}_i}(\omega) = \sigma^2_{\boldsymbol{E}_i}(\omega) \cdot \boldsymbol{I}$, therefore $\lambda_i(\omega)$ is taken as $\lambda_i(\omega) = \frac{\sigma_{\boldsymbol{E}_i}(\omega)}{\tau_i(\omega)}$.

After these simplifications, the estimation of the current is performed in just one step, by calculating the square root of the cross spectral matrices at the sources:

$$\hat{\Sigma}^{\frac{1}{2}}_{J_i}(\omega) = \boldsymbol{G} \cdot \boldsymbol{K}^t \cdot (\boldsymbol{K} \cdot \boldsymbol{G} \cdot \boldsymbol{K}^t + \lambda^2(\omega) \cdot \boldsymbol{I})^{-1} \cdot \boldsymbol{S}^{\frac{1}{2}}_i(\omega)$$

This formulation reduces the number of mathematical operations needed for the calculation of the cross spectral matrices at the sources and therefore it is useful as a practical approach to solve the problem. On the other hand, $\lambda_i(\omega)$ can be easily obtained from any procedure inverse procedure, both for real as well as for complex data.

From $\hat{\Sigma}_{J_i}^{\frac{1}{2}}(\omega)$ the cross spectral matrices at the sources can be obtained with the following equation:

$$s_{i;r,s}(\omega) = \hat{\Sigma}_{J_i}^{\frac{1}{2}}(\omega)[r] \cdot \hat{\Sigma}_{J_i}^{\frac{1}{2}}(\omega)[s]^*$$

where $s_{i;r,s}(\omega)$ is the cross spectral matrix at the sources for subject i at frequency ω, between sources r and s. $\hat{\Sigma}_{J_i}^{\frac{1}{2}}(\omega)[r]$ is the row of the cross spectral square root matrix which correspond to source r.

Normative SPM with EEG source spectra

In our case, for each subject VARETA is fitted for the 3244 (Ng) sources and 49 (Nω) frequencies, which produces 177,527 log-transformed spectral values at the sources $s_{i;r,r}(\omega)$. These values are considered as individual variations around an age dependent populational mean:

$$\log(s_{i;r,r}(\omega)) = \mu_r(\omega, age) + \varepsilon_r(\omega)$$

The population mean value for the log spectrum at voxel r $\mu_r(\omega, age)$ is a (usually nonlinear) function of age and the error term $\varepsilon_r(\omega)$ is assumed to be Gaussian with standard deviation $\sigma_r(\omega, age)$. In our qEEGT toolbox, these mean $\mu_r(\omega, age)$ values were obtained from the Cuban Human Brain Mapping Normative EEG database. This database covers an age range from 5 to 87 years old. The study was performed in a stratified manner, increasing the sample size in those ages where the age variation is known to be higher, i.e., from 5 to 25 years old. Polynomials heteroscedastic regression equations up to second order were calculated for each frequency and source (lead). The order of the polynomial in each case was obtained using AIC (Szava et al., 1994).

The Z-transform for the source spectrum of any voxel (lead) is defined as:

$Z_{i;r,r}(\omega) = \frac{\log(s_{i;r,r}(\omega)) - \mu_r(\omega,age)}{\sigma_r(\omega,age)}$ where $Z_{i;r,r}(\omega)$ is the Z transform of the source log spectrum for individual *i* at voxel r.

Moreover, the correction by the already mentioned Global Scale Factor (GSF) makes our qEEGT analysis compatible with many different recording systems. It has been shown that our normative database can be successfully used with recordings obtained from different systems, attenuating the differences between them due to calibration, amplifiers and other technical aspects.

Appendix II: The qEEGt interface

To be able to run the qEEGt plugin in CBRAIN, the user first needs an account to have access to the system. This step is accomplished at the CBRAIN portal (https://portal.cbrain.mcgill.ca), using the option "Request account". Once having the credentials, the process to calculate the qEEGt is as follows:

a) login into the CBRAIN portal

b) creates a project (Figure 1). If the project is already created, then just select it.

c) upload a dataset in an accepted format by the qEEGt, which can be done via the web-based interface or through a secured FTP server. Data should be compressed as a tar.gz file.

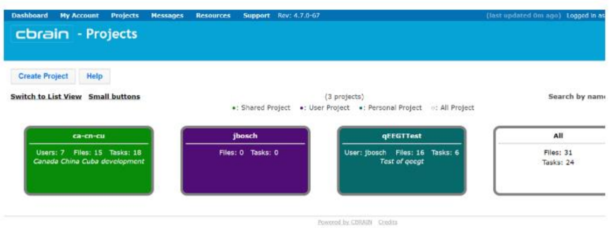

Figure 1: CBRAIN main user interface at login.

d) with the dataset selected, launch a new task (Figure 2). A window appears showing all available pipelines.

e) Inside "Software Package" the QEEGt tool must be selected. The "qeeg" plugin will appear at the right side and it must also be selected.

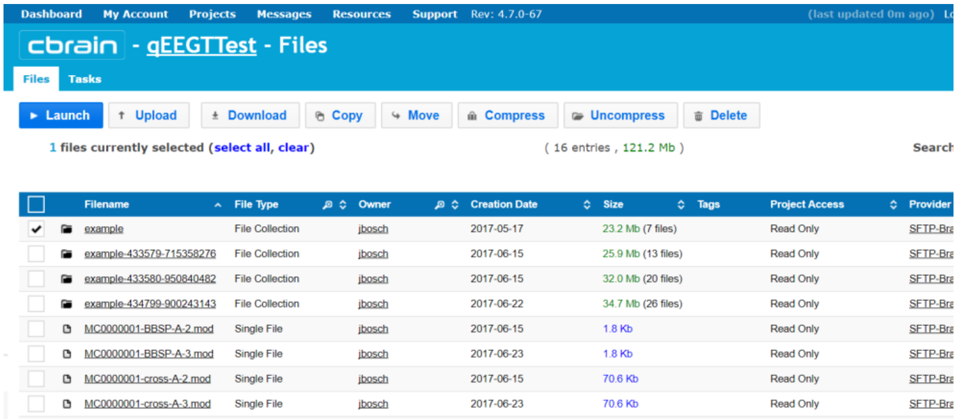

Figure 2: qEEGt task interface showing the data

f) After selecting the "qeeg" plugin, two options appear, one for the user to select the server where to run the plugin (which is optional) and the second one is to specify the desired qEEGt parameters ("Prepare qeeg").

g) The qEEG panel will appear immediately. Figure 3 shows the interface of the qEEGt-plugin in CBRAIN, with all the available user options. There are three input file formats currently available: one for the newly defined extension of the Brain Imaging Data Structure to handle EEG data (BIDS-EEG), one specific for the Neuronic PLG format created by the Cuban Neuroscience Center; and a third one for an ASCII file in a specific format, which is described in Appendix III.

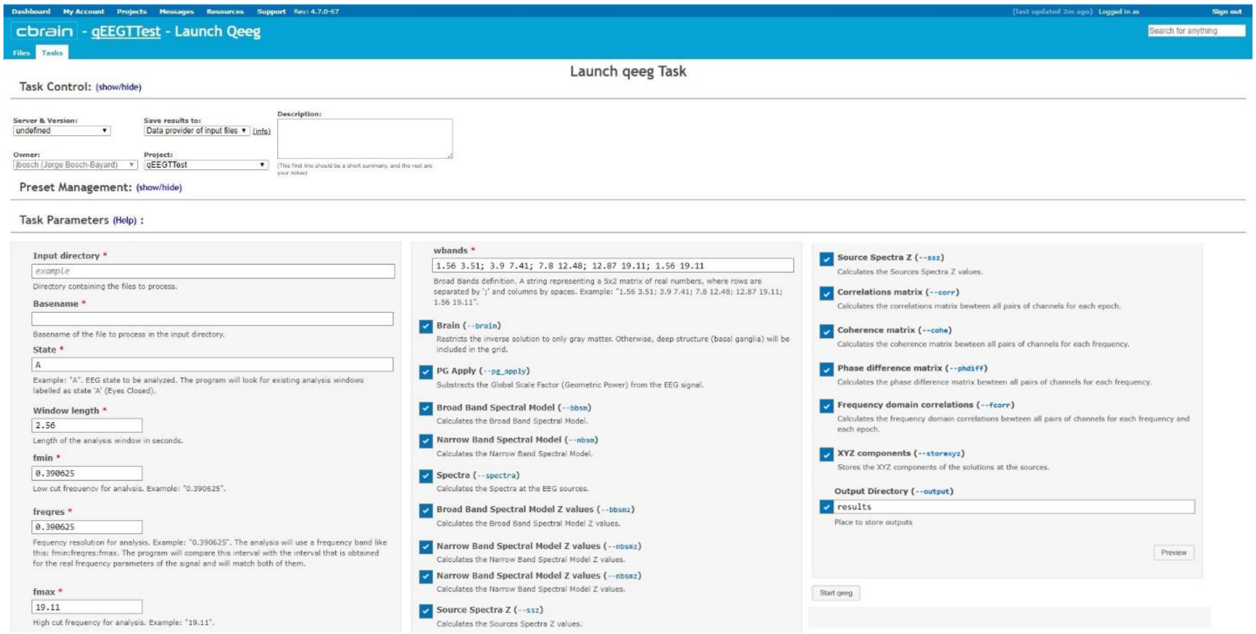

Figure 3: Main interface of the qEEGT plugin in CBRAIN. The necessary parameters for qEGT calculations are defined here.

h) Once the user has selected the appropriate options for the pipeline, including the input directory, input file and output directory, must click on the option “Start qeeg” option. CBRAIN launches the pipeline on remote resources, manages the execution and provide monitoring of the launched task. Once finished, places the results to any location in the CBRAIN ecosystem that the user specified.

To have a fast view at the results and produce the outputs shown in **Error! Reference source not found.**, **Error! Reference source not found.**, **Error! Reference source not found.** and more the user only needs to enter the results directory, the figures will start to be showing and the user obtain a new one by clicking on the results files.

Ease of use

We have measured the ease of use of the qEEGt-plugin for CBRAIN. For this purpose, we selected 12 first and second-year Master students in Biomedical Engineering from the University of Electronic Science and Technology of China. These students did not have any prior exposure either to CBRAIN or to

the qEEGT toolbox, were not fluent in English, and used a network connected to CBRAIN with a bandwidth of 10 Mbits/second. We obtained, from the time logs of CBRAIN, the time each student used first to calculate the qEEGT measures and later, in a second session, to inspect the results and load the qEEGt results images as shown in Figure 4. As shown in Figure 9, the median time for qEEGt processing was 15 minutes with a range from 9 to 26 minutes. This dispersion of processing time depended critically on the length of the que for the process queue. Logging in again and visualizing results had a median duration of 2 minutes and a dispersion from 1 to 5 minutes.

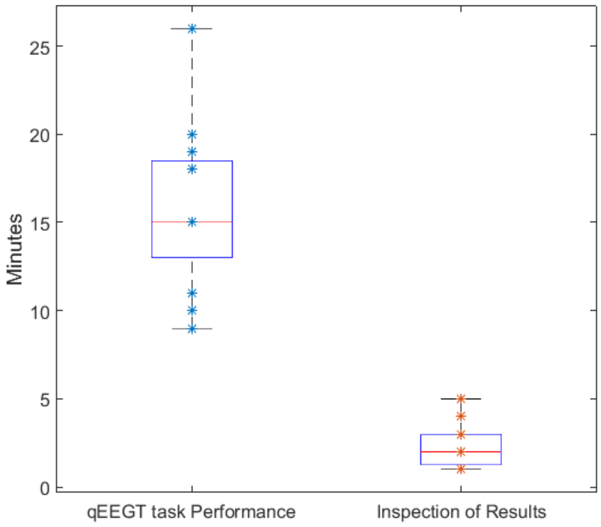

Figure 4. Distribution of times in minutes for carrying out qEEG processes and for inspection of results.

Appendix III: Accepted EEG formats for the qEEGT toolbox

ASCII format

EEG data saved in text format is accepted, with the following format:

```
NAME=Jane Doe
 SEX=F
 AGE=20.94
 SAMPLING_FREQ=200
 EPOCH_SIZE=512
 NCHANNELS=19
 MONTAGE=
 Fp1-REF
 Fp2-REF
 F3_-REF
…
```

and so on. The program expects NCHANNELS lines with their names. AFTER THE CHANNELS NAMES, THE EEG DATA COMES BY LINES where each line is an instant of time and each column represents a channel. If the EEG contains 30 segments of 512 points each and 19 channels, then

30*512 lines of 19 columns of numbers (either float or integer) are expected.

References

Babiloni, C., Barry, R. J., Başar, E., Blinowska, K. J., Cichocki, A., Drinkenburg, W. H. I. M., … Hallett, M. (2019). International Federation of Clinical Neurophysiology (IFCN) – EEG research workgroup: Recommendations on frequency and topographic analysis of resting state EEG rhythms. Part 1: Applications in clinical research studies. *Clinical Neurophysiology*. https://doi.org/10.1016/j.clinph.2019.06.234

Biscay Lirio, R., Valdés Sosa, P. A., Pascual Marqui, R. D., Jiménez-Sobrino, J. C., Alvarez Amador, A., & Galán Garcia, L. (1989). Multivariate Box-Cox transformations with applications to neurometric data. *Computers in Biology and Medicine*, *19*(4), 263–267. https://doi.org/10.1016/0010-4825(89)90013-9

Bosch-Bayard, J., Valdés-Sosa, P., Virues-Alba, T., Aubert-Vázquez, E., John, E. R., Harmony, T., … Trujillo-Barreto, N. (2001). 3D statistical parametric mapping of EEG source spectra by means of variable resolution electromagnetic tomography (VARETA). *Clinical EEG (Electroencephalography)*, *32*(2), 47–61. https://doi.org/10.1177/155005940103200203

Brillinger, D. R. (1974). *Time series: data analysis and theory*. Holt, Rinehart, and Winston.

Casanova, R., Valdes-Sosa, P., Garcia, F. M., Aubert, E., Riera, J. J., Korin, W., & Lins, O. (2000). Frequency Domain Distributed Inverse Solutions. In *Biomag 96* (pp. 189–192). New York, NY: Springer New York. https://doi.org/10.1007/978-1-4612-1260-7_45

Dempster, A. P., Laird, N. M., & Rubin, D. B. (1977). Maximum Likelihood from Incomplete Data via the EM Algorithm. *Journal of the Royal Statistical Society. Series B (Methodological)*. WileyRoyal Statistical Society. https://doi.org/10.2307/2984875

Evans, A., Collins, D., Millst, S., Brown, E., Kelly, R., & Peters, T. (1993). 3D statistical neuroanatomical models from 305 MRI volumes. In *Proceedings of IEEE- Nuclear Science Symposium and Medical Imaging Conference* (pp. 1813–1817). Retrieved from http://www.citeulike.org/user/nguizard/article/7253872

George, J. S., Lewis, P. ., Schlitt, H. ., Kaplan, L., Gorodnitski, I., & Wood, C. . (1995). Strategies for source space limitation in tomographic inverse procedures. In C. Baumgartner, L. Deecke, S. Gerhard, & S. J. Williamson (Eds.), *Proc. 9th Int. Conf. On Biomagnetism* (pp. 357–362). Vienna: Elsevier, Amsterdam.

Hernández, J. L., Valdés, P., Biscay, R., Virues, T., Szava, S., Bosch, J., … Clark, I. (1994). A global scale factor in brain topography. *The International Journal of Neuroscience*, *76*(3–4), 267–278. Retrieved from http://www.ncbi.nlm.nih.gov/pubmed/7960483

Mardia, K. V., Kent, J. T., & Bibby, J. M. (1997). *Multivariate analysis*. (Z. W. Birnbaum & E. Lukacs, Eds.) (6th ed.). London: Academic Press.

Pascual-Marqui, R D, Michel, C. M., & Lehmann, D. (1994). Low resolution electromagnetic tomography: a new method for localizing electrical activity in the brain. *International Journal of Psychophysiology : Official Journal of the International Organization of Psychophysiology*, *18*(1), 49–65. Retrieved from http://www.ncbi.nlm.nih.gov/pubmed/7876038

Pascual-Marqui, Roberto D. (2007). Discrete, 3D distributed, linear imaging methods of electric neuronal activity. Part 1: exact, zero error localization. Retrieved from http://arxiv.org/abs/0710.3341

Riera, J., Aubert, E., Valdés, P., Casanova, R., & Lins, O. (1996). Discrete Spline Electric-Magnetic Tomography (DSPET) based on Realistic Neuronatomy. In *Proceedings of The Tenth International Conference on Biomagnetism, BIOMAG'96*. Santa Fe, New Mexico: Ed. C. Wood.

Szava, S., Valdes, P., Biscay, R., Galan, L., Bosch, J., Clark, I., & Jimenez, J. C. (1994). High resolution quantitative EEG analysis. *Brain Topography*, *6*(3), 211–219. Retrieved from http://www.ncbi.nlm.nih.gov/pubmed/8204408

Tarantola, A. (1987). *Inverse problem theory : methods for data fitting and model parameter estimation*. Elsevier.

Valdes-Sosa, P., Garcia, F., & Casanova, R. (1996). Variable Resolution Electromagnetic Tomography. In *Proceedings of The Tenth International Conference on Biomagnetism, BIOMAG'96*.

Valdés, P., Bosch, J., Grave, R., Hernandez, J., Riera, J., Pascual, R., & Biscay, R. (1992). Frequency domain models of the EEG. *Brain Topography*, *4*(4), 309–319. Retrieved from http://www.ncbi.nlm.nih.gov/pubmed/1510874
