# Supplementary material for: A Quantitative EEG Toolbox for the MNI Neuroinformatics Ecosystem: Normative SPM of EEG Source Spectra
Source: Front Neuroinform. 2020 Aug 7;14:33. doi: 10.3389/fninf.2020.00033 (PMC7427620; doi:10.3389/fninf.2020.00033)
Supplement: Supplementary file 2 [file Table_1.DOCX]

# How to manage the norms coefficients of the Cuban Human Brain Mapping Project (CHBMP)

The current normative regression equations of the Cuban Human Brain Mapping Project, in its present version, are calculated for the following datasets:

- two brain states: Eyes Closed (EC) and Eyes Open (EO)
- two montages: Average Reference (AVR) and Record Montage (REC)
- two data scale options: Global Scale Factor (GSF) spectra corrected and RAW spectra (RAW)
- two models: Narrow Band (NB) and Broad Band (BB)
- two Measures: Scalp and Sources
- two Head Models: only Gray Matter (GM) and Gray Matter + Basal Ganglia (BG)

The table below summarizes the file names with the normative regression coefficients and the normative type in each case:

| File Name | State | Montage | Correction | Model | Measure | Cortex |
| --- | --- | --- | --- | --- | --- | --- |
| A_AVR_PG_BB_.NRM | EC | AVR | GSF | BB | Scalp | - |
| A_AVR_PG_ET_BG_GF.NRM | EC | AVR | GSF | NB | Sources | BG |
| A_AVR_PG_ET_GM_GF.NRM | EC | AVR | GSF | NB | Sources | GM |
| A_AVR_PG_NB_.NRM | EC | AVR | GSF | NB | Scalp | - |
| A_AVR_RD_BB_.NRM | EC | AVR | RAW | BB | Scalp | - |
| A_AVR_RD_ET_BG_GF.NRM | EC | AVR | RAW | NB | Sources | BG |
| A_AVR_RD_ET_GM_GF.NRM | EC | AVR | RAW | NB | Sources | GM |
| A_AVR_RD_NB_.NRM | EC | AVR | RAW | NB | Scalp | - |
| A_REC_PG_BB_.NRM | EC | REC | GSF | BB | Scalp | - |
| A_REC_PG_NB_.NRM | EC | REC | GSF | NB | Scalp | - |
| A_REC_RD_BB_.NRM | EC | REC | RAW | BB | Scalp | - |
| A_REC_RD_NB_.NRM | EC | REC | RAW | NB | Scalp | - |
| B_AVR_PG_BB_.NRM | EO | AVR | GSF | BB | Scalp | - |
| B_AVR_PG_ET_BG_GF.NRM | EO | AVR | GSF | NB | Sources | BG |
| B_AVR_PG_ET_GM_GF.NRM | EO | AVR | GSF | NB | Sources | GM |
| B_AVR_PG_NB_.NRM | EO | AVR | GSF | NB | Scalp | - |
| B_AVR_RD_BB_.NRM | EO | AVR | RAW | BB | Scalp | - |
| B_AVR_RD_ET_BG_GF.NRM | EO | AVR | RAW | NB | Sources | BG |
| B_AVR_RD_ET_GM_GF.NRM | EO | AVR | RAW | NB | Sources | GM |
| B_AVR_RD_NB_.NRM | EO | AVR | RAW | NB | Scalp |  |
| B_REC_PG_BB_.NRM | EO | REC | GSF | BB | Scalp | - |
| B_REC_PG_NB_.NRM | EO | REC | GSF | NB | Scalp | - |
| B_REC_RD_BB_.NRM | EO | REC | RAW | BB | Scalp | - |
| B_REC_RD_NB_.NRM | EO | REC | RAW | NB | Scalp | - |

The normative files contain the norm coefficients obtained by second order polynomial regressions against the logarithm of the age for each channel (at the scalp) and source generators (both for the Gray Matter segmentation as well as the Gray Matter plus Basal Ganglia segmentation) for each specific model.

For the narrow band model, the coefficients are obtained for each frequency bin from 0.39 Hz to 19.11 Hz every 0.39 Hz.

For the broad band model, the following bands definition is used:

Broad Bands Frequency Definition:

| Band | Low Freq (Hz) | High Freq (Hz) |
| --- | --- | --- |
| Delta | 1.56 | 3.51 |
| Theta | 3.9 | 7.41 |
| Alpha | 7.8 | 12.48 |
| Beta | 12.87 | 19.11 |
| Total | 1.56 | 19.11 |

The Broad Band model includes the following measurements:

- Absolute Power
- Relative Power
- Mean Frequency

To get access to the normative coefficients for each specific combination of choices, two Matlab functions are provided:

- readnrmcbe: for the Narrow Band model, i.e., at the scalp and at the sources
- readnrmcba: for the Broad Band model

Function call from either the Matlab command line or inside a Matlab function or script

- [meancoef, stdcoef, state, pgcorrect, freqres, freqsHz]=readnrmcBE(nrmbdfname,age)

Input parameters are:

- nrmbdfname: is the file name of the file containing the specific norms coefficients.
- age: the age for which the norm coefficients are required

Output parameters are:

- meancoef: a matrix with the population means for all the channels/sources and the frequency range, evaluated at the given age
- stdcoef: a matrix with the population standard deviation for all the channels/sources and the frequency range, evaluated at the given age
- state: 1- for Eyes Closed; 2 – for Eyes Open
- pgcorrect: 1 – means the spectra scaled by the Global Scale Factor; 0 – Not scaled
- freqres: Frequency resolution in Hz
- freqsHz: Vector containing the frequency bins in Hz
- [mncoefPA, stdcoefPA, mncoefPR, stdcoefPR, mncoefFM, stdcoefFM, state, pgcorrect, freqres, nrm_band_index]=readnrmcBA(nrmbdfname,age)

Input parameters are:

- nrmbdfname: is the file name of the file containing the specific norms coefficients.
- age: the age for which the norm coefficients are required

Output parameters are:

- mncoefPA: a matrix with the population means for Absolute Power for all the channels and the 5 frequency bands included in the Broad Band model, evaluated at the given age
- stdcoefPA: a matrix with the population standard deviation for Absolute Power for all the channels and the 5 frequency bands included in the Broad Band model, evaluated at the given age
- mncoefPR: a matrix with the population means for Relative Power for all the channels and the 4 frequency bands (except Total band) included in the Broad Band model, evaluated at the given age
- stdcoefPR: a matrix with the population standard deviation for Relative Power for all the channels and the 4 frequency bands (except Total band) included in the Broad Band model, evaluated at the given age
- mncoefFM: a matrix with the population means for Mean Frequency for all the channels and the 5 frequency bands included in the Broad Band model, evaluated at the given age
- stdcoefFM: a matrix with the population standard deviation for Mean Frequency for all the channels and the 5 frequency bands included in the Broad Band model, evaluated at the given age
- state: 1- for Eyes Closed; 2 – for Eyes Open
- pgcorrect: 1 – means the spectra scaled by the Global Scale Factor; 0 – Not scaled
- freqres: Frequency resolution in Hz
- nrm_band_index: indexes of the frequency bins included in each band

Examples of use:

>> age = 20;

%Reading a normative file for narrow band at the scalp

>> filename = 'A_AVR_PG_NB_.NRM';

>> [meancoef, stdcoef, state, pgcorrect, freqres, freqsHz]=readnrmcbe(filename, age);

%Reading a normative file for broad band at the scalp

>> filename = 'A_AVR_PG_BB_.NRM';

>> [mncoefPA, stdcoefPA, mncoefPR, stdcoefPR, mncoefFM, stdcoefFM, state, pgcorrect, freqres, nrm_band_index]=readnrmcba(filename,age);

%Reading a normative file for narrow band at the sources

>> filename = 'A_AVR_PG_ET_GM_GF.NRM';

>> [meancoef, stdcoef, state, pgcorrect, freqres, freqsHz]=readnrmcbe(filename,age);
